# Infertility and Unrealized Ideal Family Size

**DOI:** 10.1111/padr.70043

**Published:** 2026-01-12

**Authors:** Ester Lazzari, Eva Beaujouan

**Affiliations:** Department of Demography, University of Vienna, 1010 Vienna, Austria; Wittgenstein Centre for Demography and Global Human Capital ( IIASA, OeAW, University of Vienna), 1010 Vienna, Austria

## Abstract

Research indicates that people often end their childbearing years with fewer children than they had expected in young adulthood. However, our understanding of the role of infertility in explaining this discrepancy remains limited. Using data from 10 low-fertility countries included in the second round of the Generations and Gender Survey, this study examines the correspondence between ideal and actual family size among men and women, as well as the influence of infertility and socioeconomic factors on whether they achieved the number of children they considered ideal for themselves. The results show that up to half of men and women end their reproductive years wishing they had more children. Having experienced infertility stands out as a key predictor of this gap, increasing the likelihood of underachieving one’s ideal family size by 17 percent and 26 percent among childless men and women, and by 12 percent and 19 percent among those with one child.

## Introduction

In advanced industrialized societies, many individuals conclude their child-bearing years having had fewer children than they had wished for in early adulthood (Berrington and Pattaro 2014; Beaujouan and Berghammer 2019; Guzzo and Hayford 2023). Within this body of research, there is a growing focus on identifying the underlying causes of this discrepancy. One potential aspect that has received limited research attention so far is the role of medical infertility. While often discussed as a residual mechanism (e.g., Nitsche and Hayford 2020), how the experience of infertility is related to completed family size in general (Greil et al. 2024) or, more specifically, to having fewer children than ideal, is rarely measured.

Reproductive capacity declines with age among women and, to a lesser extent, men (Eijkemans et al. 2014; Brandt et al. 2019). Given the trend toward delaying family formation, infertility—medically defined as the inability to achieve pregnancy after 12 or more months of unprotected intercourse—is likely to play an increasingly important role in people’s ability to realize a desired family size. This issue, therefore, warrants focused study. Further support for the notion that an increasing share of individuals are falling short of their childbearing aspirations due to infertility is reflected in the increasing utilization of assisted reproductive technology (ART) treatments, especially among older women and couples (Ben Messaoud, Bouyer, and de La Rochebrochard 2020; Lazzari, Gray, and Chambers 2021; Lazzari and Tierney 2023; Tierney and Cai 2019). However, ART is not always successful, and success rates decline substantially with age (Newman, Paul, and Chambers 2022). As a result, for a number of men and women postponing childbearing, the experience of infertility likely remains an unresolvable challenge to the realization of a desired family size.

Using the second round of the Generations and Gender Survey (GGSII), this paper explores the extent to which people do not realize their personal fertility ideals and the factors involved in this process, with a particular focus on the role of infertility. We take a different approach from previous studies by measuring the discrepancy between ideals and outcomes based on individuals’ ideal family size reported near the end of their re-productive years, rather than at the beginning. Our analysis begins by calculating the proportion of individuals who meet, exceed, or fall short of their retrospective personal fertility ideals in each country, and by examining how these outcomes are associated with the experience of infertility. Then, we use logistic regressions to study the association between the realization of these ideals and self-reported infertility, while also controlling for factors reflecting life course events or structural constraints to childbearing, such as education and relationship trajectories. All analyses are conducted separately by sex and by parity (0, 1, or 2+ children).

## Background

### Assessing the discrepancy between fertility preferences and outcomes

Previous literature has measured the discrepancy between fertility preferences and actual fertility outcomes using both aggregate- and individual-level data. A potential pitfall when using aggregate data is the risk of misinterpreting population-level measures as reflections of individual outcomes. In some cases, the aggregate discrepancy may appear smaller than it actually is if over- and under-achievement offset each other to some extent (Harknett and Hartnett 2014; Beaujouan and Berghammer 2019). For example, Quesnel-Vallée and Morgan (2003) showed that the seemingly high agreement between fertility intentions and actual behavior in the United States masks substantial individual-level discrepancies due to compensatory errors, meaning that many women did not achieve their intended family size. Because similar dynamics may be at work in other countries, a comprehensive understanding of the issue requires analyses that move beyond aggregate-level comparisons and consider individual-level data.

Individual-level research has been conducted in specific countries. These studies adopt either a short-term perspective, examining how fertility intentions align with outcomes three to four years later (e.g., Spéder and Kapitány 2009; Régnier-Loilier and Vignoli 2011) or cover a longer term perspective, comparing intentions expressed in early adulthood with completed family size (Quesnel-Vallée and Morgan 2003; Morgan and Rackin 2010; Berrington and Pattaro 2014; Nitsche and Hayford 2020; Guzzo and Hayford 2023). This scholarship consistently demonstrates that in today’s high-income countries, a substantial proportion of individuals do not realize their earlier fertility plans.

Measuring fertility preferences at younger ages presents several technical challenges. Some individuals, particularly those with lower education levels, may already have children and be at more advanced life stages (e.g., married and in employment) compared with those with higher education, complicating direct comparisons. Furthermore, “don’t know” responses are common. This uncertainty aligns with the theoretical understanding of fertility as a process of discovery unfolding over individuals’ reproductive lives and thus should be recognized as a valid response rather than a mere measurement error (Trinitapoli and Yeatman 2018; Ní Bhrolcháin and Beaujouan 2019).

Studies looking at preferences in early adulthood often use a measure of fertility intentions rather than desires, because intentions imply a commitment to act, whereas desires are more abstract (Miller and Pasta 1993; Ajzen and Klobas 2013). Still, Rackin and Bachrach (2014) demonstrated that fertility intentions expressed in early adulthood often reflect general perceptions of ideal families rather than concrete, realistic plans, and therefore may be less meaningful for both policy and research.

Later in life, fertility targets tend to adjust. This may be due to evolving life aspirations, as people reach socially relevant milestones to family formation, such as entering a lasting relationship (Hayford 2009), or find competing interests that take precedence over childbearing (Carmichael and Whittaker 2007). Difficulties conceiving or other health constraints may also lead people to revise their plans (Gray, Evans, and Reimondos 2013; Yeatman, Sennott, and Culpepper 2013). In that case, a significant life goal may remain unfulfilled. Because both evolving aspirations and changing circumstances can influence fertility decisions, it is challenging to isolate those whose parenting desires were ultimately unmet.

To account for some of these fluctuations in preferences, we propose using a measure of personal ideal family size collected at the end of the reproductive years. We use the term unrealized fertility to denote the gap between the ideal and actual number of children, both measured at ages 42–50. This approach helps identify individuals who would have preferred to have more children than they ultimately did, filtering out midlife changes in intentions. Relatively few studies in the existing literature use a measure of fertility ideals collected at the end of the reproductive period to estimate unrealized fertility, and those who have done so focus on non-Western contexts (Channon and Harper 2019; de Carvalho, Wong, and Miranda-Ribeiro 2016; Casterline and Han 2017; Assaf and Moonzwe Davis 2022).

### Integrating infertility into the picture of unrealized fertility

Infertility has not been treated as a distinct mechanism, hindering the achievement of fertility goals. Instead, it is often seen as a residual factor, implicitly accounted for by the age effect, with no previous study providing direct evidence on the importance of infertility for unrealized fertility. A study exploring the link between infertility and completed family size showed that individuals who experience infertility do not necessarily have fewer children (Greil et al. 2024). Another study found that among women and men with a strong short-term intention to have a child, the odds of doing so within four years did not significantly differ according to their perceived ability to conceive (Beaujouan et al. 2019). One possible explanation for these results is that experiencing infertility may signal a particularly strong commitment to parenthood, as those who try more intensely also have more opportunities to encounter infertility. However, as infertility often results in an individual being unable to have (all) the children they wanted, we suggest that a larger proportion of individuals who report an ideal family size greater than the one they ultimately achieved have experienced infertility compared with the general population.

Research suggests that people tend to remember their past fertility intentions in a way that is consistent with their actual reproductive outcomes (Trinitapoli and Yeatman 2018). In particular, people with self-reported infertility appear to have a distinctive relationship with their reproductive goals. Individuals who self-report infertility tend to express stronger and more persistent fertility desires (Shreffler et al. 2016; Lazzari 2025). Therefore, they are likely to be overrepresented in the population with unrealized fertility. This is an important aspect to keep in mind as we work to understand who experiences unrealized fertility and which aspects of respondents’ histories contribute to this experience.

The correlation between infertility and unrealized fertility may vary by birth order. Difficulties conceiving are more frequently reported at lower parities (Bushnik et al. 2012; Passet-Wittig et al. 2020), and childless individuals may be especially motivated to achieve parenthood and thus be more determined to overcome infertility. Reflecting this, ART is used more often for first-order births than for subsequent ones (e.g., White and McQuillan 2006; Moreau et al. 2010). Childless individuals with unrealized fertility likely represent a distinct group, for whom unmet reproductive goals carry particular emotional weight. Parents with unrealized fertility, while wishing to have more children than they had, may nonetheless feel more satisfied than the childless. Among parents, those with one child, falling short of the two-child norm, may experience their situation differently from those with larger families. Hence, the meaning of unrealized fertility likely differs across parity groups. Accordingly, we distinguish between three groups: those who had no children despite wanting some, those who had one child but wanted more, and those who had two or more children but would have preferred to have more.

Finally, social science research rarely addresses the reproductive experiences of men (Culley, Hudson, and Lohan 2013). It is generally assumed that men perceive their reproductive trajectories differently from women and have less accurate reporting patterns and lower awareness of infertility and assisted reproduction (Daniluk and Koert 2013; Rendall et al. 1999). However, recent studies have shown that the quality of data for men can be affected by survey design but is otherwise often very good (Belgherbi and de La Rochebrochard 2018; Joyner et al. 2012). The prevalence of infertility among men is difficult to determine, but it is clear that they are emotionally affected by their childlessness and difficulties conceiving (Hadley and Hanley 2011; Culley, Hudson, and Lohan 2013). Therefore, we expect a clear relationship between infertility and unrealized fertility among men, though it may be weaker than in women due to lower awareness or prevalence of infertility.

### Other factors of unrealized fertility

A variety of individual-level factors have been shown to influence fertility behavior (Balbo, Billari, and Mills 2013). We are particularly interested in the mechanisms through which some of these factors can lead individuals to fall short of their ideal family size. Education used to have a negative impact on achieving a desired family size among women and a positive one among men (Becker 1991). However, this no longer aligns with recent empirical evidence, which shows a convergence in fertility outcomes by education among cohorts born between the late 1960s and 1970s in many high-income countries. This shift has been attributed to policies increasingly designed to support working mothers, as well as the growing involvement of partners in child-rearing, both of which have helped reduce the incompatibility between participation in the labor market and childbearing (McDonald 2013; Esping-Andersen and Billari 2015; Goldscheider, Bernhardt, and Lappegård 2015). By contrast, less-educated women may face increasing challenges in achieving their reproductive plans due to the economic uncertainty associated with their occupations and greater union instability (Hellstrand, Nisén, and Myrskylä 2024; Perelli-Harris and Amos 2015; Smock and Schwartz 2020). This suggests that the positive gradient in unrealized fertility by education may be fading or even reversing.

Childbearing is also closely linked to relationship trajectories. Individuals who are unmarried, divorced, or separated are less likely to meet their reproductive aspirations compared with those in stable marriages (Berrington and Pattaro 2014; Jalovaara and Fasang 2017; Morgan and Rackin 2010), while union dissolution has a negative impact on completed family size (Fostik et al. 2023), though re-partnering can partially offset this effect (Winkler-Dworak et al. 2017; Thomson et al. 2012), especially among men (Beaujouan and Solaz 2013). Moreover, the age at which individuals form their first union and the timing of re-partnering have a negative influence on completed family size (Fostik et al. 2023; Beaujouan and Solaz 2013; Thomson et al. 2012; Morgan and Rackin 2010; Nitsche and Hayford 2020; Quesnel-Vallée and Morgan 2003). However, the implications for unrealized fertility are less straightforward, as individuals who desire fewer children may also decide to form partnerships later in life. The limited existing literature on this topic has not found a significant association between postponement of union formation and the underachievement of fertility desires (Nitsche and Hayford 2020).

Finally, the large cross-country variation in context and social institutions suggests that unrealized fertility will vary across countries. Contexts in which policies allow for a good work–family balance and favorable economic conditions may be particularly conducive to achieving fertility targets. At the aggregate level, Beaujouan and Berghammer (2019) found large regional variation in the gap between fertility intentions at earlier ages and completed fertility, with southern and German-speaking European countries having the largest gaps and Eastern European countries the smallest. We expect to see similar patterns in the current study.

## Data

The analyses draw on data sourced from the second round of the GGS, an open-access, cross-national survey that examines family events, fertility, and gender dynamics among nationally representative samples of respondents (http://www.ggp-i.org). Data were collected between 2020 and 2023, predominantly through Computer-Assisted Web Interviewing. Compared with its predecessor, the GGSII introduced new items, including a question asking respondents up to age 60 whether they had ever experienced difficulties conceiving. In contrast, the first GGS round only asked about current difficulties and included respondents up to age 40.

As a result, GGSII is the first survey to provide retrospective, cross-national data on lifetime infertility prevalence, enabling the study of infertility experiences across individuals’ entire reproductive life course. Validation work confirms that GGSII estimates are broadly consistent with population-based data (Leocádio et al. 2023). However, the mean age at birth is slightly higher than in population statistics and, in Denmark, births appear to be underreported, leading to a higher share of childless respondents (Leocádio et al. 2023).

Data collection for the GGSII is ongoing. We restrict our analyses to the 10 countries^1^ that have released data and provided the relevant variables. Our analyses include men and women nearing the completion of their reproductive lifespans, aged 42–50 at the time of the survey (corresponding to birth cohorts 1971–1980, depending on the year of fieldwork). The sample for the descriptive analysis describing the proportion of individuals who meet, exceed, or fall short of their fertility ideals includes 4,589 men and 6,465 women who provided a numeric response to the question on ideal family size. For the logistic regression analyses, the sample with complete information (i.e., men and women who were not missing any information for either outcome or explanatory variables) decreased to 4,042 men and 5,645 women. In these 10 countries, 70 percent of respondents aged 42–50 (both sexes) had complete information, ranging from 53 percent in Argentina to 90 percent in Moldova (see Table A1 in the Online Appendix).

### Variables

#### Ideal and actual family size

Ideal family size is a numerical variable based on the question: “For you personally, what would be the ideal number of children you would like to have or would have liked to have?” This item captures personal, retrospective family size ideals and not a general societal norm, making it an informative measure of individual preferences over the life course. Some men and women did not provide a numeric response but instead answered “don’t know” or refused to answer (up to 13 percent and 7 percent, respectively, depending on the country). The absolute number and proportion of such cases are reported in Table A1 in the Online Appendix. These respondents were excluded from all analyses. A sensitivity test in which, as an extreme case, “don’t know” responses were recoded as zero ideal family size yielded consistent results with those presented in this paper (Online Appendix Table A2).

Actual family size is also a numerical variable, defined as the total number of children ever born, including both biological and adopted children.^2^ Our selection of respondents aged 42–50 allows us to reconstruct almost completed fertility for most women, as chances to give birth after age 42 are very low (although this may be less the case for men). Comparison of these two values provides an indicator of whether the actual family size is smaller than ideal.

Previous studies on the discrepancy between early fertility preferences and outcomes relied on measures of intended family size, which reflect concrete plans to have children (Miller and Pasta 1993). By contrast, fertility ideals capture desired family size, which reflects a wish for children not necessarily tied to a commitment to act (Miller and Pasta 1993). While fertility intentions become less relevant at the end of the reproductive life, when there is awareness that outcomes can no longer be changed, ideals may persist, providing insights into whether long-term preferences remained unfulfilled. For this reason, a measure of fertility ideals, rather than intentions, is well-suited to this analysis.

#### Experience of infertility

To identify men and women who have experienced infertility in their lives, we used the question: “Was there ever a time when you and a partner were trying to get pregnant but did not conceive within at least 12 months?” This aligns with the medical definition of infertility by specifying a prolonged period of unsuccessfully attempting to conceive. By definition, infertility is observable only among those who have attempted conception. Some individuals may be infertile without knowing it because they never tried to conceive, while others may meet the medical criteria but do not report infertility because they never wished to have children. Consequently, self-reported infertility captures not only a biological condition but also implies that respondents have, at some point, embraced parenthood as a desired social role (Greil, Slauson-Blevins, and McQuillan 2010).

#### Other variables

We use information on the number of unions, separations, or divorces, and age at first union to reconstruct relationship trajectories up to the time of interview. Respondents were classified as either: currently in their first union, separated from a first union and not re-partnered, in a second or subsequent union (re-partnered), or never having been in a union. In addition, we account for the age at first union formation. Marital and cohabiting unions are not distinguished, as previous research suggests that union stability, rather than union type, is more critical for childbearing outcomes (Thomson et al. 2012). Educational attainment is categorized as tertiary or below tertiary. This measure reflects the career opportunities and workplace conditions experienced during respondents’ reproductive years, as well as the expectations and social norms that may shape fertility aspirations and decisions. Descriptive statistics for all these measures can be found in Table A3 in the Online Appendix.

## Methods

We begin by describing the mean ideal and actual family size among individuals aged 42–50. This provides context for the study and serves as a benchmark for comparison with other studies reporting the discrepancy between ideal and actual family sizes at the aggregate level (e.g., Testa 2012; Beaujouan and Berghammer 2019). Given our interest in the constraints on childbearing, we then examine the disparity between ideal and actual family sizes at the individual level by reporting the proportion of respondents who, by ages 42–50, have had fewer children than ideal. These results are tabulated by parity to provide insight into the preferred number of children at the individual level.

We then explore the factors influencing the realization of an ideal family size. We start with a descriptive overview of the mean ideal and actual family size among respondents who have and have not experienced infertility. We then employ multivariate logistic regression models to examine the correlates of unrealized fertility, stratifying by parity and sex. The dependent variable in this analysis is binary, with a value of 1 assigned to individuals with a lower actual than ideal family size. Those who have either met or exceeded their ideal family size are combined into a single reference category. Although multinomial logistic regression would theoretically be more appropriate for analyzing the three potential outcomes of the dependent variable, this approach was not used due to the small size of the group exceeding their ideal family size. Combining these categories facilitates the interpretability of the results and aligns with the primary focus of the analysis, which is to characterize those who fall short of their ideal family size.

Two models are used to estimate the likelihood of having fewer children than ideal by age 42–50, applied to three groups: those who are childless, those with one child, and those with two or more children. The first model captures the bivariate association between infertility and unrealized fertility. The second model includes infertility and the independent variables of age at first union formation, relationship history, and educational attainment. This approach allows us to first show the unconditional association between infertility and the outcome and, then, to explore whether other variables alter this association.

## Results

### Descriptive results

**Figure 1:**
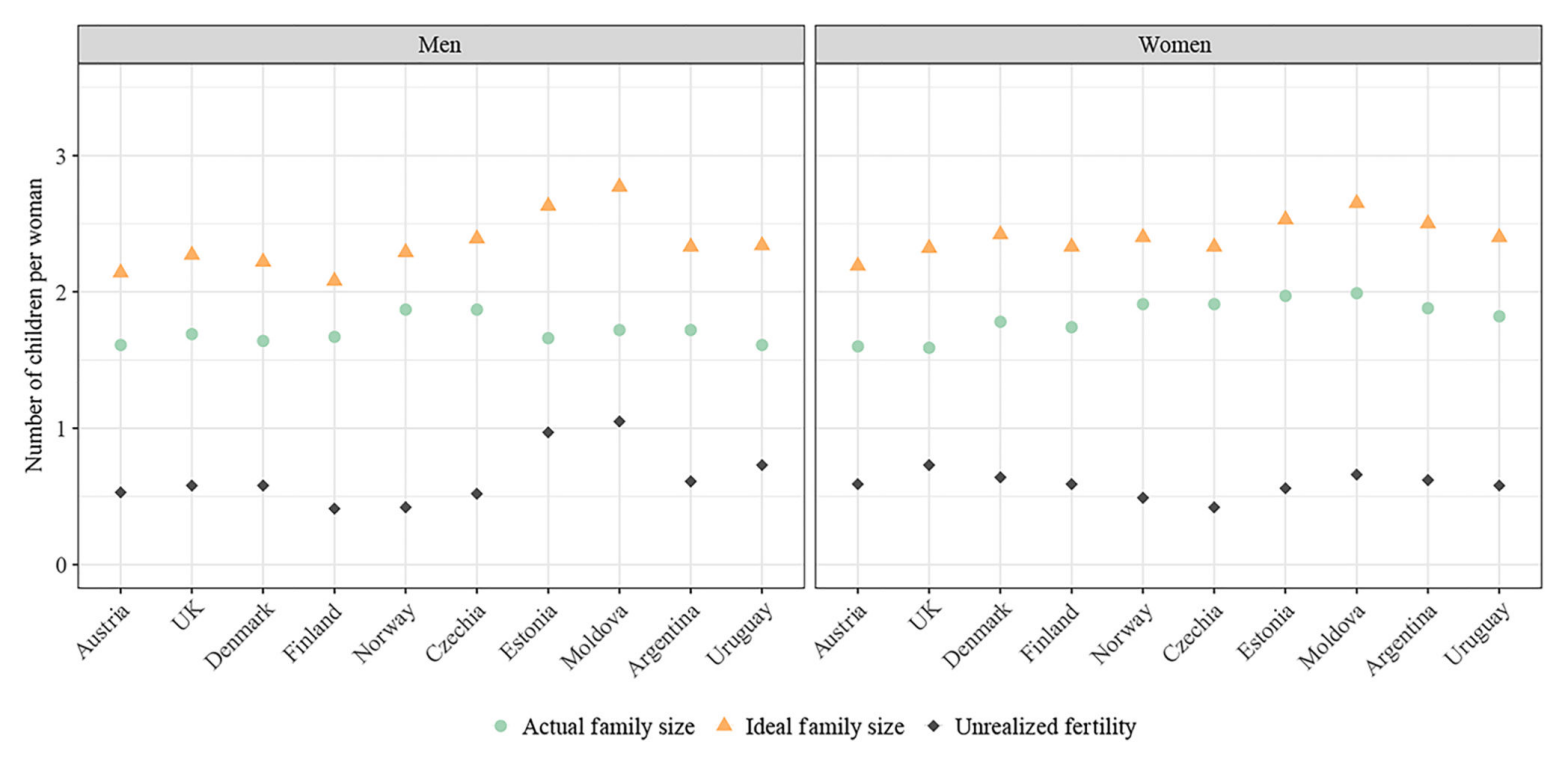
Mean retrospective ideal and actual family size by age 42–50 by sex and country SOURCE: Authors’ calculations based on GGSII data.

Figure 1 presents descriptive information on retrospective ideal and actual family size at age 42–50 across the 10 countries in Europe and Latin America. Among women, the mean ideal family size ranges from 2.19 children per woman in Austria to 2.65 in Moldova, while for men it spans from 2.08 in Finland to 2.77 in Moldova. Despite ideals being above two children, the mean actual number of children in all countries falls below two, resulting in a clear gap between the two measures. For women this gap varies from 0.42 in Czechia to 0.73 in the United Kingdom, whereas for men it ranges from 0.41 in Finland to 1.05 in Moldova. In six of the countries analyzed, the gap is larger for women than for men. However, it is important to note that men aged 42–50 have not yet completed their reproductive years, meaning the gap for them could be somewhat overestimated. Women generally report higher fertility ideals than men, although in Czechia and Moldova, fertility ideals are higher among men than among women.

**Figure 2:**
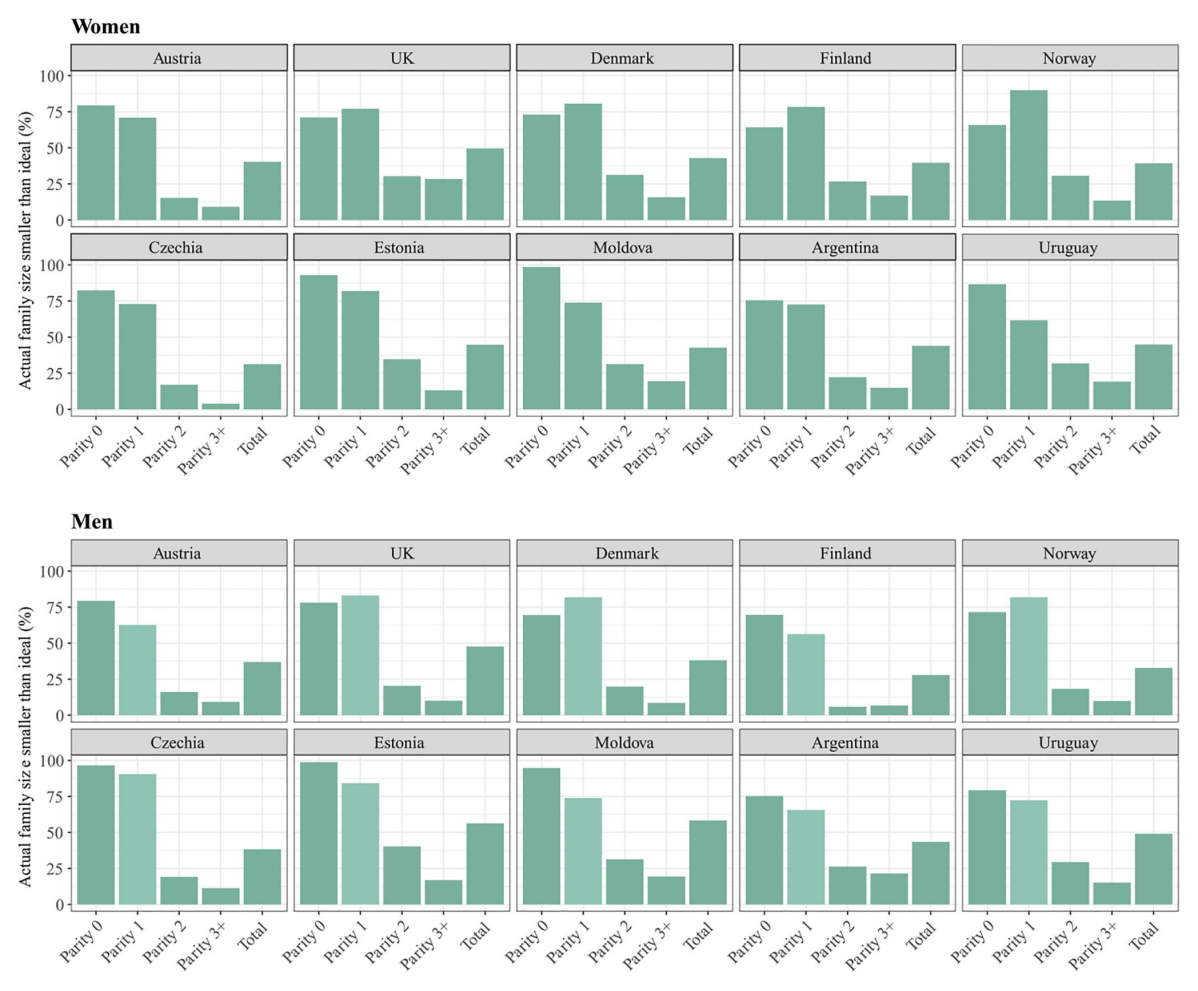
Percentage of respondents with an actual family size smaller than their retrospective ideal by age 42–50 by sex, country, and parity SOURCE: Authors’ calculations based on GGSII data.

Figure 2 shows the proportion of individuals who have reported a retrospective ideal family size larger than their actual number of children. Between one-fourth and half of men and between one-third and half of women expressed a preference for having more children than they had. By contrast, the proportion of individuals who had more children than their ideal is very low in all countries, ranging from 2 percent to 6 percent among men and 2 percent to 4 percent among women (not shown).

The parity breakdown presented in Figure 2 demonstrates that respondents with two or three children report a low discrepancy with their ideal family size. Among childless respondents, the vast majority would have ideally preferred to have children (64–99 percent of women and 69–99 percent of men). Childless men and women in the Nordic countries and the United Kingdom show a higher acceptance of their childless state compared to other countries. This suggests that, at the individual level, childlessness in these countries is less often experienced as an unwanted outcome. High levels of unrealized fertility are, however, observed among individuals with only one child.

**Figure 3:**
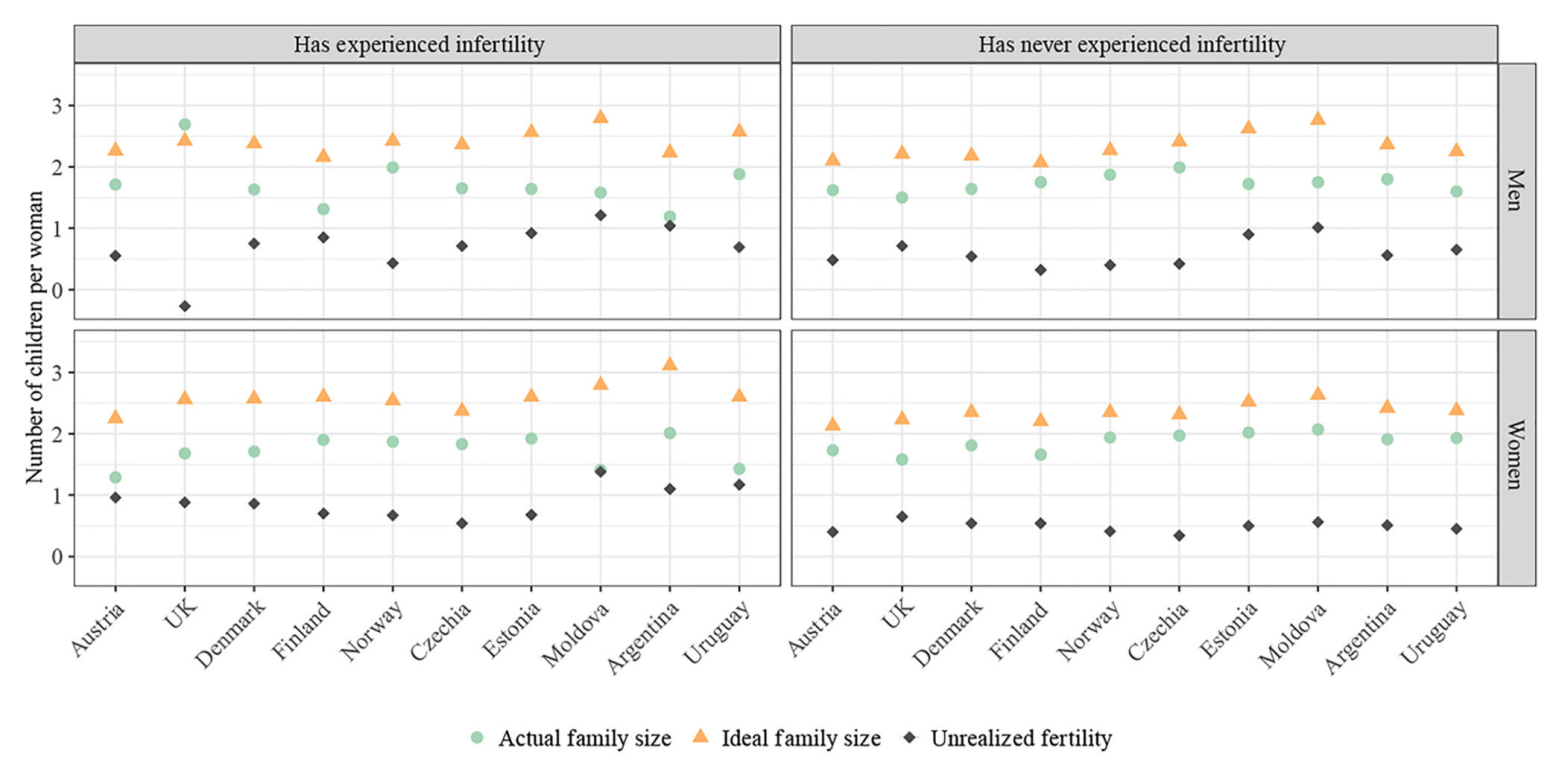
Mean retrospective ideal and actual family size by age 42–50 by infertility status, sex, and country SOURCE: Authors’ calculations based on GGSII data.

We then analyzed the mean ideal and actual family sizes among respondents with and without a history of infertility (Figure 3). Across all countries, women who had experienced difficulties conceiving consistently reported higher ideal family sizes but lower or comparable actual family sizes compared to those without such experiences.^3^ Consequently, the fertility gap was always larger among women who had experienced difficulties conceiving. For men, the findings were broadly consistent with those for women, with a few exceptions (e.g., in the United Kingdom). However, given the relatively small sample size of men who reported infertility, these results should be interpreted with caution.

### Logistic regression analyses

Table 1 shows the proportion of men and women who reported infertility, by whether or not they realized their ideal family size. Across the total sample and within all parity subgroups, those who fell short of their ideal number of children are consistently more likely to have experienced infertility in their lives. For men who experienced infertility, the difference between those that realized and those that did not realize their fertility ideals is modest in the overall sample (17.3 percent vs. 22.5 percent) and at parity two or above (18.3 percent vs. 20.0 percent). However, the contrast is larger at parity 0 (5.1 percent vs. 18.1 percent) and parity 1 (16.1 percent vs. 29.5 percent). For women, the contrast is sharper. About one in three women with unrealized fertility (32.3 percent) reported infertility, compared to only one in five (18.1 percent) among those who realized their ideals. The difference is particularly pronounced at lower parities: 32.0 percent versus 4.9 percent at parity 0 and 36.8 percent versus 15.8 percent at parity 1. At parity two or above, the gap narrows to 28.6 percent versus 19.1 percent.

Results from the logistic regression models are presented in Tables 2–4 for each final parity (0, 1, and 2 or more children). A positive average marginal effect indicates a higher likelihood of ending the reproductive life with fewer children than ideal compared to the reference category. In each parity group, Model 1 shows the bivariate association between infertility and the outcome, without adjustment for other factors. Model 2 adds age at first union, relationship history, and education.

Table 2 presents the results for men and women who were childless at age 42–50. In this group, infertility emerges as a strong predictor of unrealized fertility: men and women who experienced infertility are 17 and 26 percentage points more likely to report having fewer children than their ideal, compared to those without infertility in Model 2. Among women, later union formation (at age 35 or above) and union instability (separation without repartnering or having been in three or more co-residential unions) further reinforce this association, whereas for men relationship-related factors are not significant. Tertiary education lowers the probability of unrealized fertility for men but not for women.

Looking at men and women with one child (Table 3), infertility remains a strong predictor of unrealized fertility, increasing the likelihood by 12 percentage points for men and 19 percentage points for women in Model 2. Among women separation, repartnering, and multiple unions are associated with a higher probability of unrealized fertility, while for men only repartnering is associated with unrealized fertility. Education has no significant effect in this group, while age at first union shows a small effect only for men.

Table 4 focuses on men and women with two or more children. In this group, the association between infertility and unrealized fertility is no longer significant for men but remains significant for women, though at a reduced magnitude. For women later union formation, multiple unions, and tertiary education are associated with unrealized fertility, while for men later first union and tertiary education significantly predict unrealized fertility.

Statistically significant gender differences were tested using interaction terms in a combined model including both men and women (results not shown). Overall, the impact of infertility is similar across genders, with the exception of the parity 2+ group, where the association is significantly stronger for women than for men. Likelihood ratio tests confirm that including infertility as an explanatory variable significantly improves model fit, with the sole exception of men with two or more children.

### Interpretation and limitations

Since the data used in this study were collected from respondents aged 42–50, variables such as the number of births, age at first cohabiting union, and relationship history rely on retrospective reports and are hence prone to recall bias, although fertility reports from GGSII have been shown to be mostly consistent with population-based estimates (Leocádio et al. 2023). The infertility variable also relies on self-reporting and, given the personal and distressing nature of infertility, individuals who refused to answer may have been more likely than average to have experienced it. Since the percentage of refusals is very small (2.6 percent of the sample), the impact of non-response bias is likely negligible.

It is also difficult to determine whether individuals with both infertility and unrealized fertility had fewer children than ideal because of infertility or due to other reasons, particularly because there is no information on the timing of the infertility episode. Some individuals who experienced infertility may have eventually overcome it but still had fewer children than desired due to other constraints. By definition, only childless respondents who reported infertility and expressed a positive ideal number of children were unable to overcome infertility despite actively trying to conceive for over a year. For this group, childlessness and unrealized fertility can be directly attributed to medical infertility.

As with any measure of fertility preferences, reporting biases—such as recall or hindsight bias—may affect estimates of unrealized fertility. Because fertility preferences can evolve over time, the number of children respondents say they ideally want may not reflect their expectations at earlier points in life. As discussed earlier, individuals who experienced infertility may be less likely to retrospectively downscale their fertility ideals compared to those whose underachievement resulted from more controllable factors (e.g., prioritizing career or adjusting to a partner’s lower desire). This interpretation aligns with recent evidence showing that men and women are unlikely to reduce their fertility desires after experiencing infertility (Lazzari 2025). Such persistence of high ideals among those who have experienced infertility could lead to an overestimation of the fertility gap associated with infertility.

Finally, the prevalence of infertility appears to vary widely across countries, and it remains unclear whether this variation reflects differences in childbearing postponement, economic contexts, or reporting patterns. Although infertility is measured using a common medical definition, its recognition may still be shaped by social context (Greil, Slauson-Blevins, and McQuillan 2010). Cross-national variation may therefore reflect not only demographic factors, such as postponement, but also cultural differences in how infertility is perceived, acknowledged, and disclosed. These differences limit the comparability of results across countries.

## Discussion

This study contributes to the growing body of literature on the factors that shape the realization of reproductive plans by highlighting the significant role played by infertility alongside other life-course and structural constraints. While most previous literature has focused on socioeconomic and cultural factors, infertility has been overlooked as a distinct determinant and, instead, treated as a residual mechanism.

In contrast with most previous research comparing individuals’ fertility preferences as young adults with later childbearing outcomes (Quesnel-Vallée and Morgan 2003; Morgan and Rackin 2010; Berrington and Pattaro 2014; Beaujouan and Berghammer 2019; Nitsche and Hayford 2020; Guzzo and Hayford 2023), we measured unrealized fertility retrospectively at the end of individuals’ reproductive lives (cf. Casterline and Han 2017). This approach allows for an analysis of the factors influencing the discrepancy between individuals’ ideal and actual number of children across the entire reproductive life course. Such analyses are rare, as studies linking fertility outcomes with early-life preferences typically rely on shorter observation periods, given that longitudinal data seldom span women’s entire reproductive trajectory (Guzzo and Hayford 2023).

Although individuals’ recollections of past childbearing preferences may be influenced by their actual childbearing experiences, our results show that a substantial share of individuals (up to one in two, depending on the country) reach the end of their reproductive lives wishing they had more children. In most low-fertility countries, the two-child family norm remains strong (Sobotka and Beaujouan 2014). In line with this norm, childlessness and having only one child are often unwanted outcomes, with around three-quarters of people in these categories not realizing their ideal family size. This pattern is remarkably stable across countries, with only minor variations. For example, childless men and women are most likely to have unrealized fertility in all countries except the Nordic countries and the United Kingdom, where it is more common among parents of one child.

Since our measure captures personal retrospective fertility ideals and not just a general societal norm, the observed discrepancy likely reflects a sense of disappointment regarding one’s final family size. This is important as unfulfilled reproductive aspirations may negatively impact individuals’ well-being, lead to distress, and lower life satisfaction (White and McQuillan 2006; McQuillan, Torres Stone, and Greil 2007).

Infertility plays a significant role in the discrepancy between individuals’ ideal and actual family size. Among men, those who are childless or have one child are 17 percent and 12 percent more likely, respectively, to have unrealized fertility compared to those without infertility experience. Among women, the corresponding figures are 26 percent and 19 percent. While infertility is often treated as a residual factor, our results indicate that it is a major contributor to unrealized fertility. Infertility not only poses a barrier to entering parenthood, but it also represents a challenge in reaching one’s ideal family size among parents.

Relationship history and age at first union emerged as important factors influencing the discrepancy between ideal and actual family size among women with no or one child and parous men. Those who formed relationships later in life or experienced union disruptions, such as separation, were more likely to have a larger discrepancy between their ideal and actual family size. The importance of the timing of union formation suggests that delayed fertility is part of the wider equation between infertility and unrealized fertility, but we lack a good indicator of fertility postponement. Integrating age at first attempt to conceive to future surveys would help to further disentangle the biological effects of aging from the broader social influences on fertility outcomes (Beaujouan et al. 2025).

Higher education was not systematically associated with a greater discrepancy. This is in contrast with earlier studies showing that highly educated women are less likely to achieve their fertility intentions (Berrington and Pattaro 2014; Beaujouan and Berghammer 2019), but in line with recent evidence showing a narrowing gap in fertility differentials by education. We found a positive association between education and unrealized fertility to hold only for men and women wishing to have large families (more than two children). For childless men, we also found that higher education was associated with a lower probability of unrealized fertility.

A key strength of this analysis lies in using a measure of ideal family size collected at age 42–50. Ending one’s reproductive window still wishing to have had more children provides valuable insight into the extent to which reproductive aspirations remain unfulfilled and how individuals cognitively frame their experiences, identifying as someone who did not achieve their ideal family size. At the same time, it is important to acknowledge the implications of using ideal family size measured at older ages. Some individuals may retrospectively adjust their stated ideals to align more closely with their actual fertility outcomes. This form of post-rationalization may serve to justify life choices or to foster a sense of acceptance about how life unfolded. For example, individuals who had fewer children than originally desired may revise their ideal family size downward, while those with unplanned births may retrospectively increase their ideal to accommodate those outcomes. Such adjustments can lead to a better match between ideals and outcomes. Although our indicator is representative of those who did not adjust their ideal family size to their number of children, it is possible that those who experienced certain life events were more willing to adjust their ideals than others. This could lead to an underrepresentation of the importance of certain social or circumstantial life factors and to an overrepresentation of others, such as infertility.

For the first time, this study clearly establishes that individuals who experience reproductive difficulties are more likely to have fewer children than their personal ideal. While this association does not allow us to infer causality, it suggests that biological constraints contribute to unrealized fertility, or at least that those who report unrealized fertility at the end of their reproductive life are often those who had experienced infertility. Given the trend toward delayed childbearing, infertility is likely to become an increasingly important factor in the discrepancy between ideal and actual family size. Hence, policies aimed at mitigating its impact could play a crucial role in supporting individuals in achieving their reproductive aspirations. This includes raising awareness of age-related decline in reproductive capacity and minimizing financial and structural barriers to infertility treatment (Fauser et al. 2024). Moreover, policies that support work–family balance—such as paid parental leave and affordable childcare—may further support individuals in realizing their ideal family size by facilitating earlier family formation.

## Supplementary Material

Supplemental Materials

## Figures and Tables

**Table 1:** Percentage of men and women according to unrealized fertility and experience with infertility by age 42–50

		Unrealized fertility ideals			Realized fertility ideals	
		Ever Experienced infertility - No	Ever Experienced infertility - Yes		Ever Experienced infertility - No	Ever Experienced infertility - Yes
Men	Parity 0	81.9	18.1		94.9	5.1
	Parity 1	70.5	29.5		83.9	16.1
	Parity 2 or above	80.0	20.0		81.7	18.3
	Total	77.5	22.5		82.7	17.3
Women	Parity 0	68.0	32.0		95.1	4.9
	Parity 1	63.2	36.8		84.2	15.8
	Parity 2 or above	71.4	28.6		80.9	19.1
	Total	67.7	32.3		81.9	18.1

SOURCE: Authors’ calculations based on GGSII data.

**Table 2:** Average marginal effects (AMEs) from logistic regression predicting unrealized fertility among childless men and women

		Men						Women			
	Model 1			Model 2			Model 1			Model 2	
	AMEs	*p*-value		AMEs	*p*-value		AMEs	*p*-value		AMEs	*p*-value
Ever experienced infertility											
No (Ref.)											
Yes	0.159	***		0.166	***		0.247	***		0.260	***
Age at first union											
25 or less (Ref.)											
26–30				0.045						0.077	
31–35				–0.004						0.092	
Above 35				0.046						0.168	***
Relationship history											
In first union (Ref.)											
Separated				0.015						0.120	**
Repartnered				0.006						0.017	
In 3+ union				0.005						0.084	*
Never been in a union				0.036						0.068	
Education											
Nontertiary (Ref.)											
Tertiary				–0.089	**					–0.040	
*N*	760			760			803			803	
Pseudo-*R*^2^	0.121			0.138			0.147			0.180	

NOTE: All models control for education and country. Reference category: having a larger or equal to ideal family size. SOURCE: Authors’ calculations based on GGSII data. Significance levels:

* *p <* 0.05,

** *p <* 0.01,

*** *p <* 0.001.

**Table 3:** Average marginal effects (AMEs) from logistic regression predicting unrealized fertility among men and women with one child

	Men						Women				
	Model 1			Model 2			Model 1			Model 2	
	AMEs	*p*-value		AMEs	*p*-value		AMEs	*p*-value		AMEs	*p*-value
Ever experienced infertility											
No (Ref.)											
Yes	0.110	***		0.116	***		0.181	***		0.185	***
Age at first union											
25 or less (Ref.)											
26–30				0.063						–0.005	
31–35				0.097	*					0.017	
Above 35				0.076						0.015	
Relationship history											
In first union (Ref.)											
Separated				0.058						0.083	*
Repartnered				0.079	*					0.115	***
In 3+ union				0.054						0.088	*
Never been in a union				0.069						0.074	
Education											
Non tertiary (Ref.)											
Tertiary				–0.013						<0.001	
Constant											
*N*	713			713			1,044			1,044	
Pseudo-*R*^2^	0.039			0.048			0.051			0.063	

NOTE: All models control for education and country. Reference category: having a larger or equal to ideal family size. SOURCE: Authors’ calculations based on GGSII data. Significance levels:

* *p <* 0.05,

** *p <* 0.01,

*** *p <* 0.001.

**Table 4:** Average Marginal Effects (AMEs) from logistic regression predicting unrealized fertility among men and women with two or more children

	Men						Women				
	Model 1			Model 2			Model 1			Model 2	
	AMEs	*p*-value		AMEs	*p*-value		AMEs	*p*-value		AMEs	*p*-value
Ever experienced infertility											
No (Ref.)											
Yes	0.026			0.025			0.105	***		0.103	***
Age at first union											
25 or less (Ref.)											
26–30				0.033						0.018	
31–35				0.074	**					0.039	*
Above 35				0.020							
										–0.015	
Relationship history											
In first union (Ref.)											
Separated				0.056						0.004	
Repartnered				0.040						0.026	
In 3+ union				0.068						0.049	*
Never been in a union				0.114						0.047	
Education											
Non-tertiary (Ref.)											
Tertiary				0.046	**					0.091	***
*N*	2,569			2,569			3,798			3,798	
Pseudo-*R*^2^	0.036			0.045			0.022			0.033	

NOTE: All models control for education and country. Reference category: having a larger or equal to ideal family size. SOURCE: Authors’ calculations based on GGSII data. Significance levels:

* *p <* 0.05,

** *p <* 0.01,

*** *p <* 0.001.

## Notes

1 For Argentina, the data refer to men and women residing in the city of Buenos Aires.

2 Sensitivity analyses excluding adopted children from completed family size produced results consistent with those reported here.

3 One possible explanation for the higher ideals among those with infertility is that all of them reported wanting to have at least one child. However, when restricting the comparison to respondents without an experience of infertility who also wanted to have at least one child, the difference persists, with individuals with infertility still reporting higher ideal family sizes, on average (Online Appendix Table A4).
