# Supplementary material for: Infertility and Unrealized Ideal Family Size
Source: Popul Dev Rev. Author manuscript; Available in PMC 2026 Jan 21. (PMC7618656; doi:10.1111/padr.70043)
Supplement: Supplemental Materials [file EMS212070-supplement-Supplemental_Materials.docx]

**Appendix**

**Table A1 (Appendix)**. Sample characteristics by country.

| Region | Country | Original  sample size | Don't know | Don't know  (%) | Refuse to answer | Refuse to answer  (%) | Missing | Sample size  for the descriptives | Sample size for the multivariate |
| --- | --- | --- | --- | --- | --- | --- | --- | --- | --- |
|  | *Men* | | | | | | | | |
| Western  Europe | Austria | 695 | 58 | 8 | 50 | 7 | 60 | 527 | 456 |
|  | UK | 655 | 69 | 11 | 15 | 2 | 82 | 489 | 408 |
| Northern  Europe | Denmark | 1147 | NA | NA | NA | NA | 296 | 851 | 778 |
|  | Finland | 259 | 33 | 13 | 10 | 4 | 27 | 189 | 174 |
|  | Norway | 552 | 51 | 9 | 28 | 5 | 54 | 419 | 383 |
| Central and  Eastern Europe | Czechia | 554 | 14 | 3 | 9 | 2 | 130 | 401 | 362 |
|  | Estonia | 921 | 108 | 12 | 49 | 5 | 71 | 693 | 613 |
|  | Moldova | 524 | 12 | 2 | 0 | 0 | 0 | 512 | 468 |
| Latin  America | Argentina | 161 | 10 | 6 | 4 | 2 | 7 | 140 | 91 |
|  | Uruguay | 413 | 11 | 3 | 8 | 2 | 26 | 368 | 309 |
|  | *Women* | | | | | | | | |
| Western  Europe | Austria | 1090 | 42 | 4 | 57 | 5 | 141 | 850 | 739 |
|  | UK | 988 | 57 | 6 | 32 | 3 | 110 | 789 | 657 |
| Northern  Europe | Denmark | 1245 | NA | NA | NA | NA | 299 | 946 | 866 |
|  | Finland | 330 | 33 | 10 | 5 | 2 | 22 | 270 | 245 |
|  | Norway | 687 | 43 | 6 | 16 | 2 | 131 | 497 | 456 |
| Central and  Eastern Europe | Czechia | 802 | 33 | 4 | 13 | 2 | 116 | 640 | 566 |
|  | Estonia | 1203 | 79 | 7 | 53 | 4 | 147 | 924 | 820 |
|  | Moldova | 734 | 5 | 1 | 1 | 0 | 0 | 728 | 660 |
| Latin  America | Argentina | 234 | 8 | 3 | 4 | 2 | 15 | 207 | 119 |
|  | Uruguay | 714 | 13 | 2 | 9 | 1 | 78 | 614 | 517 |

**Table A2 (Appendix)**. Ideal and actual family size, men and women aged 42-50 by country and region.

|  | **Main analysis** | | | | **Sensitivity analysis** | | |
| --- | --- | --- | --- | --- | --- | --- | --- |
| **Region** | **Country** | **Ideal family size** | **Actual family size** | **Gap** | **Ideal family size** | **Actual family size** | **Gap** |
| *Men* | | | | | | | |
| Western  Europe | Austria | 2.14 | 1.61 | 0.53 | 1.94 | 1.46 | 0.48 |
|  | UK | 2.27 | 1.69 | 0.58 | 1.95 | 1.4 | 0.55 |
| Northern  Europe | Denmark | 2.22 | 1.64 | 0.58 | 2.22 | 1.59 | 0.63 |
|  | Finland | 2.08 | 1.67 | 0.41 | 1.86 | 1.66 | 0.2 |
|  | Norway | 2.29 | 1.87 | 0.42 | 2.09 | 1.84 | 0.25 |
| Central and  Eastern Europe | Czechia | 2.39 | 1.87 | 0.52 | 2.28 | 1.64 | 0.64 |
|  | Estonia | 2.63 | 1.66 | 0.97 | 2.26 | 1.69 | 0.57 |
|  | Moldova | 2.77 | 1.72 | 1.05 | 2.71 | 1.69 | 1.02 |
| Latin  America | Argentina | 2.33 | 1.72 | 0.61 | 2.16 | 1.48 | 0.68 |
|  | Uruguay | 2.34 | 1.61 | 0.73 | 2.26 | 1.54 | 0.72 |
| *Women* | | | | | | | |
| Western  Europe | Austria | 2.19 | 1.6 | 0.59 | 2.07 | 1.51 | 0.56 |
|  | UK | 2.32 | 1.59 | 0.73 | 2.07 | 1.47 | 0.6 |
| Northern  Europe | Denmark | 2.42 | 1.78 | 0.64 | 2.42 | 1.77 | 0.65 |
|  | Finland | 2.33 | 1.74 | 0.59 | 2.1 | 1.74 | 0.36 |
|  | Norway | 2.4 | 1.91 | 0.49 | 2.23 | 1.92 | 0.31 |
| Central and  Eastern Europe | Czechia | 2.33 | 1.91 | 0.42 | 2.25 | 1.94 | 0.31 |
|  | Estonia | 2.53 | 1.97 | 0.56 | 2.33 | 1.87 | 0.46 |
|  | Moldova | 2.65 | 1.99 | 0.66 | 2.64 | 1.98 | 0.66 |
| Latin  America | Argentina | 2.5 | 1.88 | 0.62 | 2.33 | 1.65 | 0.68 |
|  | Uruguay | 2.4 | 1.82 | 0.58 | 2.4 | 1.95 | 0.45 |

Note: The sensitivity analysis assumes that respondents who answered “don’t know” to the question “For you personally, what would be the ideal number of children you would like to have or would have liked to have?” have an ideal family size of 0.

**Table A3 (Appendix)**. Descriptive statistics. Men and women aged 42-50 by country and region.

|  | Parity 0 | | Parity 1 | | Parity 2 and above | |
| --- | --- | --- | --- | --- | --- | --- |
|  | *Men* | *Women* | *Men* | *Women* | *Men* | *Women* |
| Ever experienced infertility |  |  |  |  |  |  |
| No | 84.61 | 74.22 | 72.73 | 68.07 | 81.41 | 78.57 |
| Yes | 15.39 | 25.78 | 27.27 | 31.93 | 18.59 | 21.43 |
| Relationship history |  |  |  |  |  |  |
| In first union | 19.87 | 25.16 | 43.03 | 41.79 | 62.02 | 57.09 |
| Separated | 11.71 | 11.33 | 11.52 | 13.55 | 4.99 | 7.92 |
| Repartnered | 14.74 | 18.06 | 22.73 | 24.02 | 20.94 | 23.54 |
| In 3+ union | 20.66 | 18.68 | 21.21 | 16.43 | 11.83 | 10.80 |
| Never partnered | 33.03 | 26.77 | 1.52 | 4.21 | 0.21 | 0.65 |
| Age at first union |  |  |  |  |  |  |
| 25 or less | 19.34 | 27.65 | 41.21 | 52.46 | 55.31 | 70.54 |
| 26-30 | 10.26 | 9.59 | 23.18 | 12.63 | 22.53 | 12.49 |
| 31-35 | 6.05 | 6.10 | 10.76 | 7.29 | 6.34 | 2.99 |
| 35 or above | 64.34 | 56.66 | 24.85 | 27.62 | 15.82 | 13.98 |
| Fertility postponement |  |  |  |  |  |  |
| Parent by age 35 | 0.00 | 0.00 | 69.85 | 78.03 | 87.96 | 96.28 |
| Not a parent by age 35 | 100.00 | 100.00 | 30.15 | 21.97 | 12.04 | 3.72 |
| Education |  |  |  |  |  |  |
| Non-tertiary | 58.29 | 45.83 | 60.00 | 54.31 | 52.92 | 48.91 |
| Tertiary | 41.71 | 54.17 | 40..00 | 45.69 | 47.08 | 51.09 |
| N | 760 | 803 | 713 | 1,044 | 2,569 | 3,798 |

**Table A4 (Appendix)**. Ideal and achieved family size among individuals with and without infertility, men and women aged 42-50 by country and region.

|  |  | Ideal family size | | | Actual family size | | | Gap | | |
| --- | --- | --- | --- | --- | --- | --- | --- | --- | --- | --- |
| Region | **Country** | **Fertile** | **Fertile, with ideal family size of at least one** | **Infertile** | **Fertile** | **Fertile, with ideal family size of at least one** | **Infertile** | **Fertile** | **Fertile, with ideal family size of at least one** | **Infertile** |
| *Men* | | | | | | | |  |  |  |
| Western  Europe | Austria | 2.10 | 2.23 | 2.26 | 1.62 | 1.71 | 1.71 | 0.48 | 0.52 | 0.55 |
|  | UK | 2.21 | 2.47 | 2.42 | 1.50 | 1.61 | 2.69 | 0.71 | 0.86 | -0.27 |
| Northern  Europe | Denmark | 2.18 | 2.41 | 2.38 | 1.64 | 1.80 | 1.63 | 0.54 | 0.61 | 0.75 |
|  | Finland | 2.07 | 2.26 | 2.16 | 1.75 | 1.89 | 1.31 | 0.32 | 0.37 | 0.85 |
|  | Norway | 2.27 | 2.43 | 2.42 | 1.87 | 1.99 | 1.99 | 0.40 | 0.44 | 0.43 |
| Central and  Eastern Europe | Czechia | 2.41 | 2.42 | 2.36 | 1.99 | 2.00 | 1.65 | 0.42 | 0.42 | 0.71 |
|  | Estonia | 2.62 | 2.64 | 2.56 | 1.72 | 1.72 | 1.64 | 0.90 | 0.92 | 0.92 |
|  | Moldova | 2.76 | 2.79 | 2.79 | 1.75 | 1.76 | 1.58 | 1.01 | 1.03 | 1.21 |
| Latin  America | Argentina | 2.36 | 2.53 | 2.23 | 1.80 | 1.93 | 1.19 | 0.56 | 0.60 | 1.04 |
|  | Uruguay | 2.25 | 2.40 | 2.57 | 1.60 | 1.69 | 1.88 | 0.65 | 0.71 | 0.69 |
| Women | | | | | | | |  |  |  |
| Western  Europe | Austria | 2.13 | 2.27 | 2.25 | 1.73 | 1.83 | 1.29 | 0.40 | 0.44 | 0.96 |
|  | UK | 2.23 | 2.49 | 2.56 | 1.58 | 1.76 | 1.68 | 0.65 | 0.73 | 0.88 |
| Northern  Europe | Denmark | 2.35 | 2.51 | 2.57 | 1.81 | 1.93 | 1.71 | 0.54 | 0.58 | 0.86 |
|  | Finland | 2.20 | 2.48 | 2.60 | 1.66 | 1.86 | 1.90 | 0.54 | 0.62 | 0.70 |
|  | Norway | 2.35 | 2.48 | 2.54 | 1.94 | 2.04 | 1.87 | 0.41 | 0.44 | 0.67 |
| Central and  Eastern Europe | Czechia | 2.31 | 2.37 | 2.37 | 1.97 | 2.03 | 1.83 | 0.34 | 0.34 | 0.54 |
|  | Estonia | 2.52 | 2.55 | 2.60 | 2.02 | 2.04 | 1.92 | 0.50 | 0.51 | 0.68 |
|  | Moldova | 2.63 | 2.64 | 2.79 | 2.07 | 2.08 | 1.41 | 0.56 | 0.56 | 1.38 |
| Latin  America | Argentina | 2.42 | 2.51 | 3.11 | 1.91 | 1.98 | 2.01 | 0.51 | 0.53 | 1.10 |
|  | Uruguay | 2.38 | 2.47 | 2.60 | 1.93 | 1.96 | 1.43 | 0.45 | 0.51 | 1.17 |
